# Rapid start-up and improvement of granulation in SBR

**DOI:** 10.1186/s40201-015-0188-9

**Published:** 2015-04-25

**Authors:** Sajjad Jalali, Jalal Shayegan, Samira Rezasoltani

**Affiliations:** Department of Chemical and of Petroleum Engineering, Sharif University of Technology, Tehran, Iran

**Keywords:** Aerobic granulation, Cationic polymer, Rapid granulation

## Abstract

**Background:**

The aim of this study is to accelerate and improve aerobic granulation within a Sequencing Batch Reactor (SBR) by cationic polymer addition.

**Methods:**

To identify whether the polymer additive is capable of enhancing granule formation, two SBRs (R1 and R2, each 0.15 m in diameter and 2 m in height) are used by feeding synthetic wastewater. The cationic polymer with concentration of 30 to 2 ppm is added to R2, while no cationic polymer is added to R1.

**Results:**

Results show that the cationic polymer addition causes faster granule formation and consequently shorter reactor start-up period. The polymer-amended reactor contains higher concentration of biomass with better settling ability (23% reduction in SVI_15_) and larger and denser granules (112% increase of granular diameter). In addition, the results demonstrate that the cationic polymer improve the sludge granulation process by 31% increase in Extracellular Polymer Substance(EPS) concentration, 7% increase in Specific Oxygen Uptake Rate(SOUR), 18% increase in hydrophobicity, and 17% reduction in effluent Mixed Liquor Suspended Solid(MLSS) concentration.

**Conclusions:**

Concludingly, it is found that using the cationic polymer to an aerobic granular system has the potential to enhance the sludge granulation process.

## Background

Aerobic granulation is a biological wastewater treatment which has been defined as a self-immobilization process to transform loose sludge flocs into dense granules [1]. Compared to activated sludge, aerobic granules have many advantages such as dense construction, excellent settling ability, simultaneous nutrient removal capability, high ability in repulsion of shock loading, and excellent tolerance for toxic substances [2]. These characteristics have led to numerous studies for identification of many factors which affect granule formation. The factors which have been studied include substrate composition [3,4], organic loading rate [5], shear force [6], dissolved oxygen concentration [7], settling time [8], substrate starvation [9], food to microorganism ratio [10], hydraulic retention time [11], volumetric exchange ratio [12], aspect ratio [13], pH [14] and oxygen concentration [15]. However, the mechanism of granulation is not yet clear [16]. Furthermore, instability during the start-up and operation of granulation in SBR have been observed [17,18].

The formation and stability of the aerobic granules are important for operation to succeed. An innovative method for improvement of granules’ stability is to apply a chemical additive. Some studies have investigated the effect of divalent metal ions on granule formation. In these works, it is reported that divalent metal ions, such as Ca^2+^ and Mg^2+^ could accelerate the granulation process through bridging between negatively charged groups on cell surfaces and linking extracellular polymers [19-21]. The results of these studies also showed that divalent metal ions indeed significantly decrease the time of granulation and result in better physical characteristics of granules and settling ability of biomass.

Researchers have suggested that granulation could be started by bacterial adsorption and bacterial adhesion to inert materials through physicochemical interactions. In addition, extracellular polymers excreted by bacteria, can strengthen the initial granules [21], so chemical additives whose behaviour is like extracellular polymers, can be more effective than divalent ions.

The key contribution of this paper is that a cationic polymer is used to enhance speed of granule formation and to improve granule structure. The cationic polymer is adsorbed to the cell surfaces which are typically negatively charged. Therefore their interactions can neutralize the cell surface charge and thus help the biomass aggregation like extracellular polymers. For implementing this idea, two bioreactors under the same operational conditions, but one without polymer addition (R1) and the other one with polymer addition (R2) are operated. Then, we measure operational parameters such as Chemical Oxygen Demand (COD), Mixed Liquor Suspended Solid (MLSS), Volatile Suspended Solid (VSS), Specific Oxygen Uptake Rate (SOUR), and Sludge Volume Index in 15 minutes (SVI_15_). Furthermore, we measure granule structure parameters such as Extracellular Polymer Substance (EPS), cell hydrophobicity and morphological parameters.

## Methods

### Geometry of reactors

**Figure 1:**
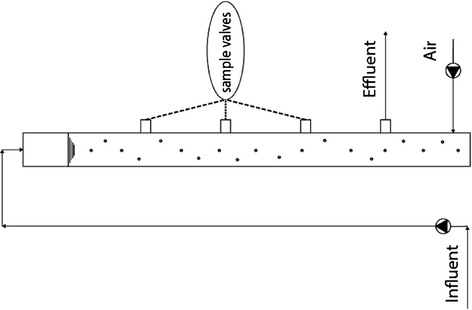
Geometry of reactors.

Diameter and height of both reactors are fifteen and two meters, respectively [13].The exit point is embedded at height of one-fifth of the reactor height which is manually controlled [12] (Figure 1). The reactor is filled by feed up to height of 1.8 m which leads to 32 liters of feed.

### Operation of reactors

Operating cycle consisted of four steps. These include aeration, sedimentation, filling and discharge. Air flowrate is set to 0.6 liters per second to maintain superficial upflow air velocity at 3.6 cm per second [6]. Optimum operation cycle is 6 hours [11], in this research, the operation cycle is chosen 12 hours because of lab limitations. Optimum settling time for aerobic granulation is five minutes [8]. For this study, the settling time is considered ten minutes at the beginning of reactor start-up. Then by improving settling ability, it gradually decreases to five minutes. The optimal discharge time is five minutes [12] which is considered in this research Filling time is chosen five minutes. Seed sludge was obtained from the wastewater treatment plant of southern Tehran. Initial biomass concentration was considered 3000 mg/L [22].

### Method of analysis

The analyses of COD, MLSS, VSS, SOUR and SVI_15_ are done according to standard methods [23]. EPS are extracted by Li’s method [24]. The polysaccharide (PS) content is determined by phenol-sulfuric acid method [25] and the protein (PN) content is measured by Lowery method [26]. Consequently, EPS value is obtained by adding PS value to PN value. Cell hydrophobicity is determined by Rosenberg et al.’s method [27] with using Hexadecane phase. The hydrophobicity is defined as the percentage of cells adhering to the hexadecane phase after 15 minutes in which water phase and hexadecane phase assumed to be in equilibrium in decantor.

Morphological parameters are determined by image processing techniques. This technique consist of taking pictures and then analysis them by Image J Software. For morphological analysis, two parameters are required: (i) Feret diameter, which is the maximum distance between two points on the perimeter of a granule’s picture, and (ii) aspect ratio, which is the ratio of maximum elliptical diameter to its minimum of granule’s picture [28]. Furthermore, granule diameter distribution is determined by sieving and image analysis.

### Feed

Synthetic wastewater is made by diluting sugar cane molasses with water to achieve COD concentration of about 1500 mg/L. Ammonium Phosphate is used to adjust values of carbon, nitrogen and Phosphorus to the 100:5:1 ratio, respectively. The pH is adjusted to 7.0 by the addition of Potassium Dihydrogen Phosphates and Potassium Hydrogen Phosphates. Cationic polymer (Reifock Flockungshilfsmittel RP3) was obtained from the Reiflock Company.

## Results and discussion

### Polymer dose

**Figure 2:**
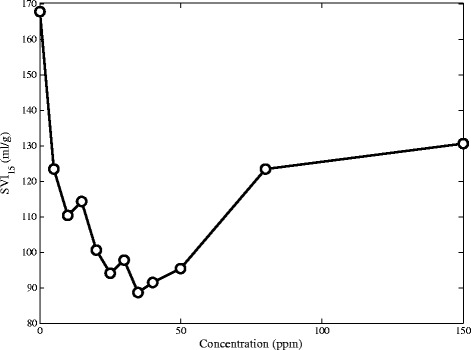
The effect of polymer concentration on SVI_15_.

Settling time and reactor exchange ratio are the most effective factors on the formation of aerobic granular sludge. SVI_15_ is a usual standard parameter that simultaneously considers these two factors. For obtaining optimum cationic polymer concentration, the changes of SVI_15_ value versus polymer concentration should be plotted. For this purpose, six three-liter cylindrical containers were filled with sludge having biomass concentration of 3000 mg/l. One container was left as a control sample without any polymer addition while different concentration of cationic polymer were added to the remaining ones. Based on Figure 2, the optimum concentration value of polymer was obtained as 30 ppm.

In low polymer concentration values up to 30 ppm, as shown in Figure 2, a significant effect on SVI_15_ can be observed, but in the moderate to high concentration values, SVI_15_ is only slightly affected. When pH is between six and eight, similar to our case, microorganisms have negative surface charge [29]. Thus, cationic polymer can easily neutralize the surface charge of cells which aggregates microorganisms to make flocs. Spatial structure of flocs is described by fractals; a mathematical concept, which is defined for irregular shape of objects that possess the property of self-similarity [30] and consists of three components: microorganisms, extra cellular polymers and water gaps. Cationic polymer probably changes fractal structure and hence decreases water gaps in flocs. With addition of polymer, the water content of flocs decreases and hence their density increases. As a result of this behaviour, settling ability of granules improves. In case of moderate to high values of polymer concentration, due to accumulation of polymer layers on surface of the flocs, repulsion forces make flocs to disintegrate. As a result, the settling ability of biomass decreases and also the supernatant liquid becomes turbid.

**Figure 3:**
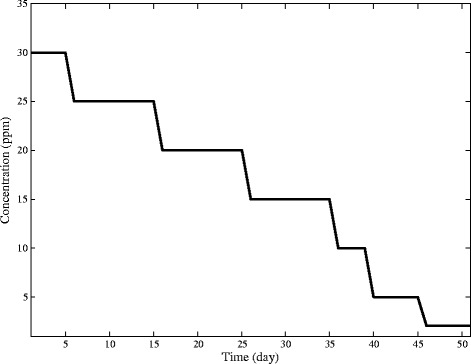
Time step for dosing polyacrylamide cationic polymer.

As the optimum concentration is equal to 30 ppm, the cationic polymer with initial concentration of 30 ppm was added to R2 and then, it decreased to 2 ppm in seven steps as shown in Figure 3.

The zeta potential of fine particles during the aerobic granulation process gradually decreased. The decrease of zeta potential might be a necessary condition for the formation and stability of aerobic granules [31]. Adding cationic polymer can decrease zeta potential during the operation of reactor.

### MLSS Concentration

**Figure 4:**
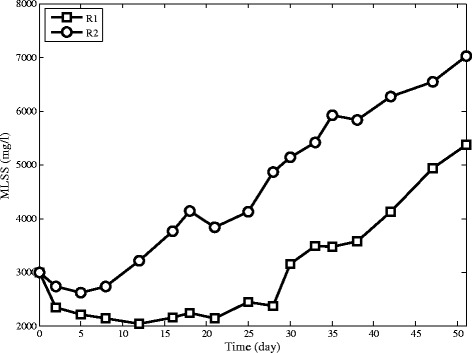
Biomass content in SBRs.

The aerobic granules appear in R2 only five days after reactor start-up, while the aerobic granules in R1 is observed after 15 days of operation .The pattern of biomass concentration variation in reactors can be divided into decreasing and increasing parts, as shown in Figure 4. In the decreasing part, both reactors lose suspended solids which could not settle within the pre-specified settling time, but the amount of reduction in R1 is more significant. In the increasing part, R2 has a considerable growth of biomass accumulation. This difference in the behaviour of the two reactors can be explained by the effect of cationic polymer on sludge characteristics which increases the rate of sludge settling and hence decreases wash out of flocs from the exiting port leading to considerable growth and less washout in R2. Finally, MLSS in R2 reaches 7100 mg/L while in R1 MLSS reaches to only 5350 mg/L. The time of MLSS which reaches its minimum in R1 and R2 are 15 and 5 days respectively which shows significant improvement of operation with using cationic polymer addition.

### Effluent suspended solid

**Figure 5:**
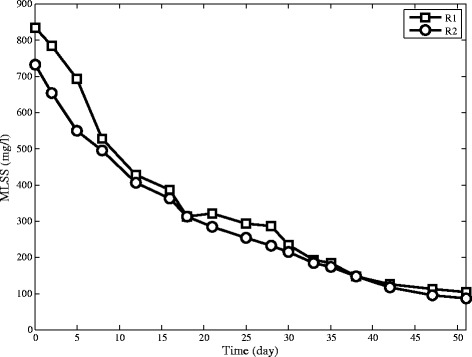
Effluent suspended solids in SBRs.

During the first days of operation, according to Figure 5, effluent suspended solids (ESS) of both reactors were high due to hindered settling regime in SBRs and high SVI_15_ of seeded activated sludge. After a few days, when granules began to form, the regime changed to free settling and hence the ESS decreased [32]. It was observed that the blank reactor lost greater amounts of biomass. This happens due to lower density and settling ability of the granules in R1 in comparison to R2.

### Volatile suspended solid

During the reactors’ operation, VSS/TSS slightly decreases in both reactors. This pattern shows that inorganic compounds increase in the granules. Inorganic compounds are dominantly polyvalent ions such as calcium, magnesium and iron which make coherent structure with extra cellular polymers and hence change granules into more durable and denser [33,34,12].

**Figure 6:**
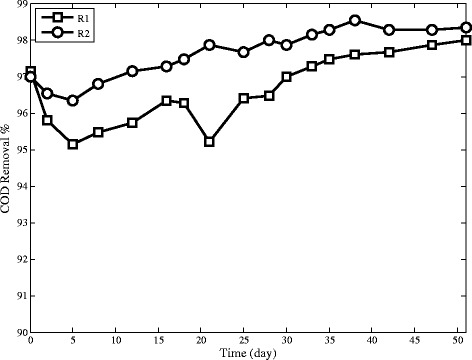
Effluent COD concentration in SBRs.

The COD removal efficiencies of both reactors are nearly constant. They almost have the same pattern over the entire time of the experiments (Figure 6).

### The settling ability of granules

**Figure 7:**
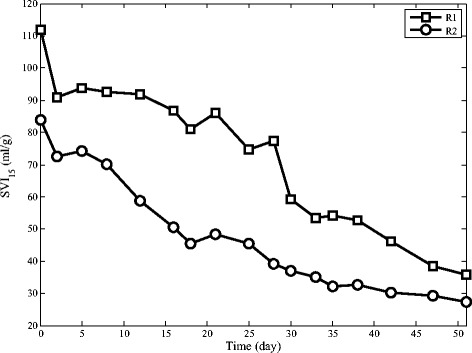
Sludge volume index in SBRs.

**Figure 8:**
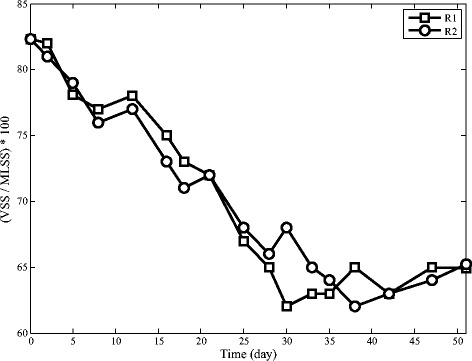
Ratio of VSS/SS in SBRs.

As shown in Figure 7, both reactors had decreasing SVI_15_ pattern. Improvement of settling ability of sludge is related to the formation of granules. Figures 8 and 7 reveal that SVI_15_ and inorganic compound concentration have a direct relationship which shows sludge settling ability improves by increasing inorganic compound contents. Although both reactors have a decreasing trend, they do not follow a similar pattern. Settling ability of granules in R2 is much better. This improvement can be explained by faster granulation in R2 and better stability of granules in this reactor. The cationic polymers may produce bridges among the negatively charged bacterial-cell surfaces through the electrostatic charge. Structure of polymer and bacterial-cell generates a construction with complex networking for enhancing bacterial aggregation which results in granules with higher density [35].

### EPS content and SOUR of granules

**Figure 9:**
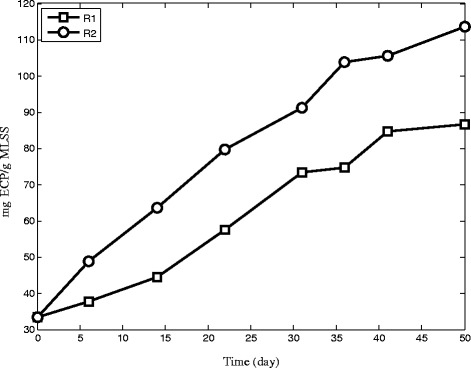
The changing pattern of EPS in R1 and R2 during operation.

EPS is considered to be essential for initiation, formation and structural stability of aerobic granules [36]. Figure 9 shows that total EPS content of granules in both reactors increase with time. This can be explained by cyclic starvation in SBR leading microbial community to produce more EPS for catching substrate [37]. The changing pattern of EPS concentration in Figure 9 shows that R2 has more EPS concentration than R1.

**Figure 10:**
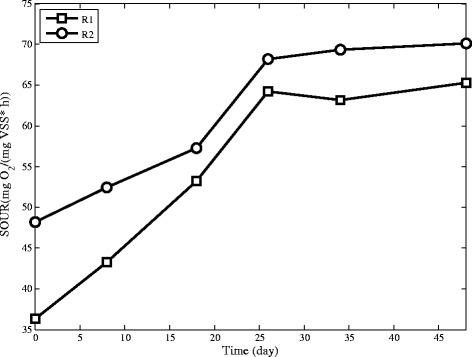
The changing pattern of SOUR in R1 and R2 during operation.

In this study, the activity of microorganisms is determined by SOUR, in terms of milligram of oxygen consumed by a gram of cell biomass per hour. The changing pattern of SOUR during the operation is shown in Figure 10, which shows that this parameter increases for both reactors. This increase can be related to granule surface and EPS enhancement [38]. Adding cationic polymer makes surface of granules rough which improves oxygen adsorption and hence the SOUR in R2 becomes more than R1 as shown in Figure 10 [39].

### Cell surface hydrophobicity

**Figure 11:**
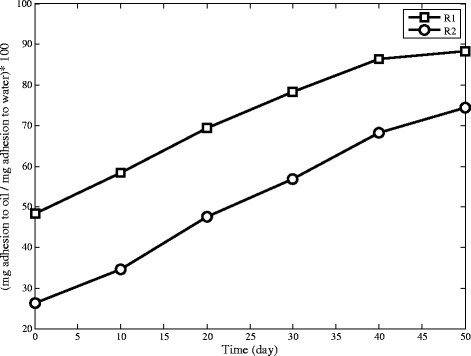
The changing pattern of hydrophobicity in R1 and R2 during operation.

During the operation, cell surface hydrophobicity significantly increases in both reactors which has been observed in [40] for non-cationic polymer reactors. Comparison between Figures 5 and 7 with Figure 11 reveals that hydrophobicity inversely depends on SVI_15_ and effluent TSS. These relationships indicate the fact that microorganisms increase their hydrophobicity in response to the cyclic starvation. As the Figure 11 shows, the hydrophobicity increases fast in the early stages of operation, but this increase slows down as the operation continues. Figure 11 also illustrates that hydrophobicity in R2 is more than R1. This can be related to the nature of cationic polymer which has hydrophobic and hydrophilic parts. Hydrophobic part of polymers attach to microorganisms and help them to achieve higher hydrophobicity [41].

### Physical properties of granules

**Figure 12:**
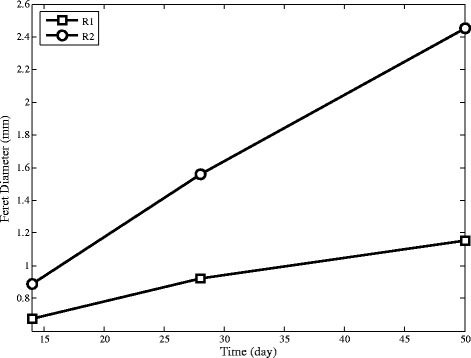
Radius of granules by image analysis in SBRs at the end of the experiment.

The size and density of aerobic granules have a profound impact on the stability and treatment performance of them. As Figure 12 shows, the size of the granules increases in both reactors, but the patterns are not the same. R2 has bigger granules than R1.

It seems that the addition of cationic polymer improves the floc formation ability of some microorganisms which could not have ability to make granules. Furthermore, this behaviour has been observed for poly aluminum chloride [42].

**Figure 13:**
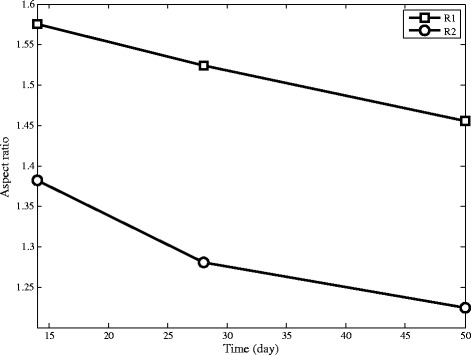
Aspect ratio of granules by image analysis in SBRs at the end of the experiment.

Figure 13 shows that granules in R2 become more spherical than R1. When granules become more spherical, they achieve greater stability and settling ability.

**Figure 14:**
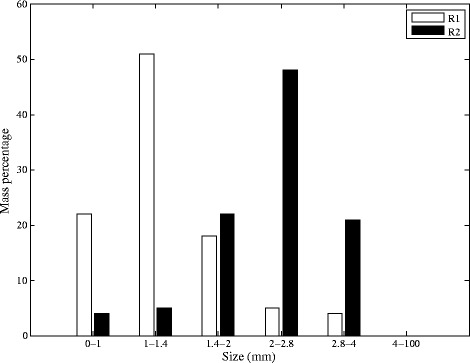
Granular size distribution by weight at the end of the experiment.

Figure 14 shows the weight distribution of granules at the end of the experiment. The maximum mass fraction of R1 is in the range of 1–1.4 mm (51% by mass), while the granules in R2 are mainly distributed in the range of 2–2.8 mm (48% by mass). Furthermore, there is a critical granular size which granules larger than that would be more vulnerable breakage [43]. Cationic polymer by making extra connections make granule more durable which can be seen by comparing between R1 and R2 in Figure 14.

## Conclusions

Formation and stability of aerobic granules is one of the most important parameters in operation and startup of aerobic granule sludge. Using chemicals for improving these characteristics has been done, but none of them is effective. In this study, we used cationic polymer as a strong flocculent agent to improve aerobic granulation. Experimental results illustrated that cationic polymer addition to the system results in fast granulation, less washout from reactor, increasing activity of microbes, more durable granules and improvement of SVI_15_.

## Abbreviations

COD: Chemical oxygen demand
ESS: Effluent suspended solid
EPS: Extracellular polymer substance
MLSS: Mixed liquor suspended solid
PN: Protein
Ppm: Part per million
PS: Polysaccharide
R1: Reactor without polymer
R2: Reactor with polymer
SBR: Sequencing batch reactor
SVI_15_: Sludge volume index
SOUR: Specific oxygen uptake rate
TSS: Total suspended solid
VSS: Volatile suspended solid
